# Supplementary material for: Circular RNA Profile in Atherosclerotic Disease: Regulation during ST-Elevated Myocardial Infarction
Source: Int J Mol Sci. 2024 Aug 19;25(16):9014. doi: 10.3390/ijms25169014 (PMC11354517; doi:10.3390/ijms25169014)
Supplement: Supplementary file 1 [file ijms-25-09014-s001.zip › ijms-3091575-supplementary.pdf]

Supplemental Figures

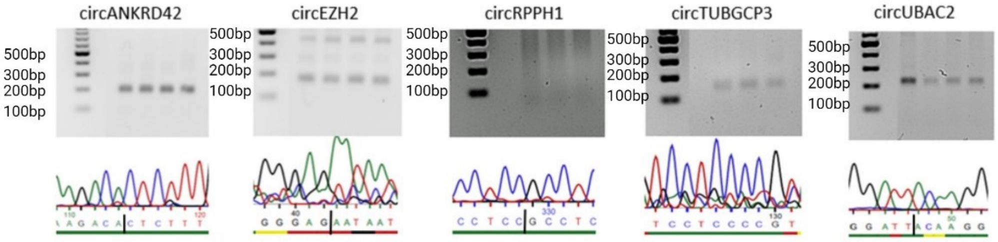

Figure S1. Gel electrophoresis of circRNA RT-qPCR products from PAXgene samples. DNA sequencing of the RT-qPCR products confirms back-splicing sites. Marker in the gels is 1 Kb ladder, and all PCR products are around 200 bp, as expected.

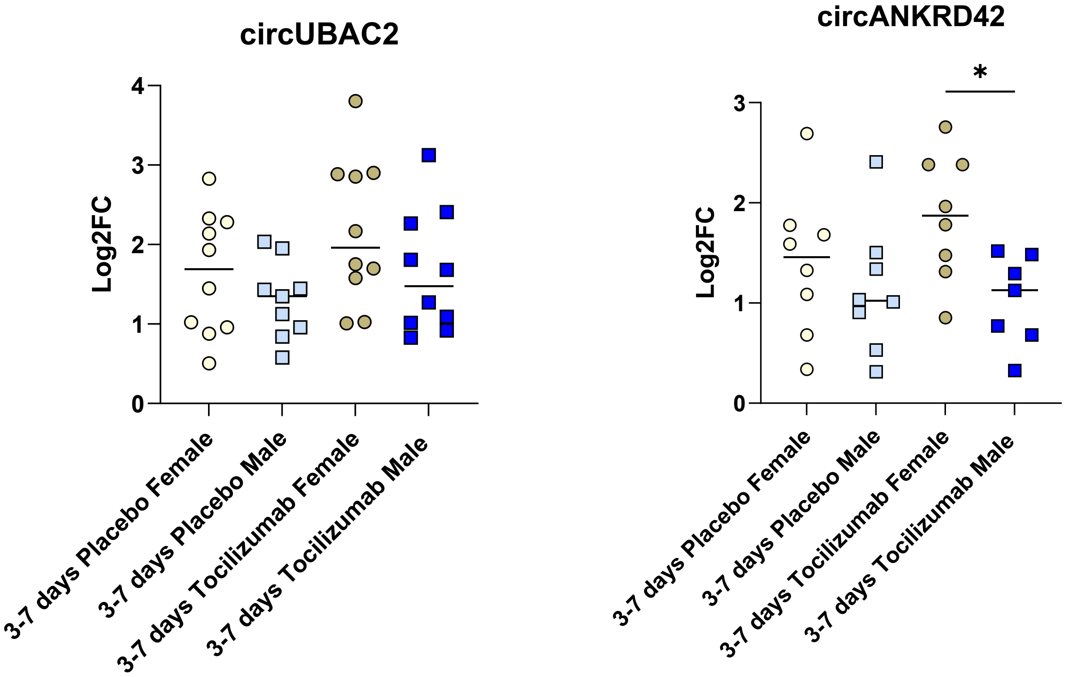

Figure S2. circRNA regulation between male and female STEMI patients randomized to placebo or tocilizumab. The figure show circRNA levels 3-7 days after hospital admission**.** *P<0.05 between females and males (unpaired T-test).

Supplemental Tables

Table S1. Primer sequences used for RT-qPCR. bp = base pair.

Genes	Primers	Size amplicon (bp)
**circRPPH1**	Has_circ_001846	200
**Forward**	5’-TTGGGAAGGTCTGAGACTAGGG-3’	
**Reverse**	5‘-CCACTGATGAGCTTCCCTCC-3’	
**circHIPK3**	Has_circ_0000284	82
**Forward**	5′-GTCGGCCAGTCATGTATCAA-3′	
**Reverse**	5′-ACCAAGACTTGTGAGGCCAT-3′	
**circANKRD42**	Has_circ_0023858	200
**Forward**	5′-GGAGAGACCCAGTGATGTGG-3′	
**Reverse**	5′-TCATCCTGGGCTGTCAGATTTGCTC-3′	
**circEZH2**	Has_circ_0006357	200
**Forward**	5’-AACCAAGAATGGAAACAGCGAAGG-‘3	
**Reverse**	5’-AGTCGCATGTACTCTGATTTT-‘3	
**circTUBGCP3**	Has_circ_0007031	200
**Forward**	5’-GGATCACATCATTGCTGCAC-‘3	
**Reverse**	5’-TGCCACAGTCGATCTGTTTT-‘3	
**circUBAC2**	Has_circ_0070720	200
**Forward**	5’-TGACTTTCTCCTCATTGAAGCTATGC-‘3	
**Reverse**	5’-TCGTTCTTGACTGCGTGAAGGTC-‘3	
**Human Beta-Actin**		
**Forward**	5’-AGGCACCAGGGCGTGAT-‘3	
**Reverse**	5’-TCGTCCCAGTTGGTGACGAT-‘3	

Table S2. Overview of top 10 up- and downregulated circRNAs in splenocytes from *ApoE*$^{-/-}$ mice. Showing FC and regulation pattern of the top 10 significantly up- and downregulated circRNAs from the array. Also shown are the workflow of all the human primers designed and why they are either included in further analysis (Y) or not (-).

	**Result from Murine circRNA Array**		**Detectible in Human Material**	
	**Name**	**Fold Change**	**Macrophages (THP-1)**	**Whole blood (PAXgene)**
**Significantly higher in ApoE$^{-/-}$**	circANKRD42	28.3	Y	Y
	circKIF21A	28.2	Y	-
	circPOR	27.2	-	-
	circSNX10	25.8	-	-
	circMYO5B	25.2	-	-
	circEZH2	23.2	Y	Y
	circCACNB2	21.1	Y	-
	circRAF1	21.0	-	-
	circCIZ1	20.3	-	-
**Significantly higher in WT**	circDOKist4	5.9	-	-
	circGAB3	5.1	-	-
	circBCKDHA	4.6	-	-
	circEPHB2	4.4	-	-
	circCALM3	4.0	-	-
	circRPPH1	4.0	-	Y
	circTUBGCP3	3.8	Y	Y
	circUBAC2	3.7	Y	Y
	circETV6	3.6	-	-

Table S3. Overview of all circRNA-miRNA-mRNA interactions investigated in RNA sequencing data. The table is showing five miRNA targets for each circRNA and five mRNA targets for each miRNA, together with data from the RNA sequencing. ND = not detected in the RNAseq.

circRNA	***miRNA-target***	***mRNA-target***	**Log2FC / Not detectible (ND)**
circUBAC2 hsa_circ_0030720	hsa-miR-200b-3p	VASH2	ND
		HIPK3	-0.35
		MAP2	ND
		ERRFI1	-1.25
		ZEB1	-0.33
	hsa-miR-2115-5p	KCNIP1	ND
		ZKSCAN2	ND

		CSNK1G3	ND
		CADM2	ND
		ZFP91	ND
	hsa-miR-509-5p	RHOT1	-0.41
		CALD1	ND
		SERTAD2	ND
		AGFG1	-0.77
		SCL38A2	ND
	hsa-miR-519d-5p	AFF4	-0.40
		IKZF2	ND
		RPP14	ND
		LPAR4	ND
		ITGA8	ND
	hsa-miR-539-3p	CREBRF	-0.70
		PDZD2	ND
		ELAVL2	ND
		ADCYAP1	ND
		TNPO1	ND
circANKRD42 hsa_circ_0023858	hsa-miR-324-5p	GAN	ND
		MGAT3	ND
		AP1G1	-0.54
		CRISP1	ND
		VDAC1	0.40
	hsa-miR-136-5p	PPP2R2A	ND
		SGIP1	ND
		MTMR4	ND
		SLAMF7	0.70
		GCNT2	ND
	hsa-miR-374a-3p	PLPPR4	ND
		RBM27	ND
		PIK3CG	-0.63
		C12ORF50	ND
		ADCYAP1	ND
	hsa-miR-330-5p	CEP85	ND
		GPD2	ND
		LETM2	-1.30
		PALM	ND
		YBEY	ND
	hsa-miR-653-3p	ZDHHC17	-0.58
		YWHAG	0.27
		ONECUT2	ND
		VEZT	0.42
		LACC1	0.91
